# Integrating TCGA and single-cell sequencing data for colorectal cancer: a 10-gene prognostic risk assessment model

**DOI:** 10.1007/s12672-023-00789-x

**Published:** 2023-09-13

**Authors:** Di Lu, Xiaofang Li, Yuan Yuan, Yaqi Li, Jiannan Wang, Qian Zhang, Zhiyu Yang, Shanjun Gao, Xiulei Zhang, Bingxi Zhou

**Affiliations:** 1 Department of Gastroenterology, Henan Provincial People’s Hospital, People’s Hospital of Zhengzhou University, School of Clinical Medicine, Henan University, Zhengzhou, 450003 China; 2 School of Basic Medicine, Zhengzhou University, Zhengzhou, 450001 China; 3 Henan Provincial Key Medical Laboratory of Genetics, Institute of Medical Genetics, Henan Provincial People’s Hospital, Zhengzhou, 450003 China; 4 Microbiome Laboratory, Henan Provincial People’s Hospital, People’s Hospital of Zhengzhou University, Zhengzhou, 450003 China

**Keywords:** Colorectal cancer, scRNA-seq, Bioinformatics, TCGA, 10-gene signature

## Abstract

Colorectal cancer represents a significant health threat, yet a standardized method for early clinical assessment and prognosis remains elusive. This study sought to address this gap by using the Seurat package to analyze a single-cell sequencing dataset (GSE178318) of colorectal cancer, thereby identifying distinctive marker genes characterizing various cell subpopulations. Through CIBERSORT analysis of colorectal cancer data within The Cancer Genome Atlas (TCGA) database, significant differences existed in both cell subpopulations and prognostic values. Employing WGCNA, we pinpointed modules exhibiting strong correlations with these subpopulations, subsequently utilizing the survival package coxph to isolate genes within these modules. Further stratification of TCGA dataset based on these selected genes brought to light notable variations between subtypes. The prognostic relevance of these differentially expressed genes was rigorously assessed through survival analysis, with LASSO regression employed for modeling prognostic factors. Our resulting model, anchored by a 10-gene signature originating from these differentially expressed genes and LASSO regression, proved adept at accurately predicting clinical prognoses, even when tested against external datasets. Specifically, natural killer cells from the C7 subpopulation were found to bear significant associations with colorectal cancer survival and prognosis, as observed within the TCGA database. These findings underscore the promise of an integrated 10-gene signature prognostic risk assessment model, harmonizing single-cell sequencing insights with TCGA data, for effectively estimating the risk associated with colorectal cancer.

**Supplementary Information:**

The online version contains supplementary material available at 10.1007/s12672-023-00789-x.

## Introduction

Human health is threatened by colorectal cancer (CRC), a highly significant gastrointestinal disease. According to a 2018 epidemiological study, it ranks fourth in terms of morbidity and fifth in terms of mortality worldwide [1]. Furthermore, CRC was identified as the second leading cause of death by 2020 [2]. There is a rising global incidence of CRC, with the projected number of cases and deaths expected to reach 2.2 million by 2030 [3, 4]. The current primary treatments for CRC include surgery, chemotherapy, radiotherapy, and targeted therapy. Additionally, owing to advancements in tumor research, immunotherapy is becoming increasingly prevalent [5]. Consequently, patient survival rates are on the rise. This positive trend is attributed to the availability of multiple treatment options as well as early CRC screening. Notably, the survival rate for patients diagnosed with CRC in its early stages is nearly 90%, compared with just 14% for those diagnosed with advanced CRC [6, 7]. The early screening and diagnosis of CRC primarily depend on techniques such as gastrointestinal endoscopy and pathological analysis, which significantly rely on the personal expertise of medical professionals. Thus, there arises an imperative to establish a molecular diagnostic technique capable of predicting the risk of CRC.

Single-cell sequencing technology is an effective tool that can reveal tumor heterogeneity and evolutionary processes at the single-cell level [8]. This technology has found applications in many tumor studies, including those involving CRC [9]. Single-cell sequencing makes it feasible to identify key genes and signaling pathways that drive tumor formation and progression. Such insights hold substantial significance for the development of novel prognostic markers and personalized treatment strategies [10].

However, prevailing research predominantly concentrates on investigating tumor heterogeneity through single-cell sequencing technology, with relatively less emphasis on its potential for prognosis assessment and personalized treatment [11]. Moreover, current prognostic models primarily rely on clinical-pathological characteristics and select known genes associated with prognosis. Yet, the predictive accuracy and clinical applicability of these models still require refinement [12]. Consequently, there is a pressing need to construct novel prognostic models grounded in more comprehensive and precise molecular markers. These models aim to enhance the precision of prognosis evaluation and the efficacy of personalized treatment [13].

At present, the principal methodologies for uncovering CRC molecular markers encompass gene expression profile analysis, genome-wide association studies (GWAS), and single-cell sequencing [14]. However, these approaches possess inherent limitations. For instance, gene expression profile analysis and GWAS typically demand a substantial sample size, often failing to capture the intricate heterogeneity of tumors [15]. By contrast, while single-cell sequencing can unveil tumor heterogeneity, the analysis of its data is intricate and necessitates specialized experimental equipment and techniques [16].

In this study, we adopted a novel approach that amalgamates single-cell sequencing with machine learning algorithms. This approach aims to comprehensively and accurately uncover molecular markers of CRC [17]. Our method not only elucidates tumor heterogeneity but also identifies genes with a strong correlation to prognosis, thereby enhancing the precision of prognosis evaluation and the efficacy of personalized treatment. Furthermore, our methodology exhibits potential for broader applications, encompassing diverse tumor types and presenting promising prospects.

## Materials and methods

### Data download and data processing

We used the Seurat package to extract single-cell sequencing data from NCBI;s GEO database, specifically targeting six primary tumor samples encompassing 6 instances of primary CRC, six liver metastases, and three Peripheral Blood Mononuclear Cell (PBMCs). Across these six samples, a total of 25,120 genes and 55,042 cells were observed. To ensure data quality, we computed the content of mitochondria and rRNA within each cell using the Performance Feature Set function. Cells were filtered based on nFeature_RNA (gene expression count) between 500 and 7000, with the exclusion of the maximum and minimum 1% of percentages. Cells were further filtered based on Ribo content (rRNA content in the cell) to maintain percent.mt (mitochondria) below 35%, and nCount_RNA (UMI count in cells) greater than 1000. Following these steps, we identified the top 2000 hypervariable genes using FindVariableFeatures and then subjected them to principal component analysis (PCA) to reduce the high-dimensional data into a low-dimensional format. We retained the top 30 principal components using ElbowPlot, utilizing a resolution of 0.1 for cluster analysis through FindAllMarkers and screening of differentially expressed genes. The subpopulations were annotated using SingleR.

We procured FPKM data and clinical information from TCGA-COAD, comprising 456 tumor samples and 41 normal samples, among which 435 samples featured survival time and status information. FPKM data underwent filtration and logarithmic transformation.

### WGCNA

To predict the scores of each sample within TCGA dataset concerning cell subpopulations, we utilized the cibersort function from the CIBERSORT package. The Pearson correlation coefficient was calculated to determine the distance of each gene from the others. Establishing a weighted co-expression network, we chose an eight-point soft threshold, followed by filtering the co-expression module using the R software package WGCNA. Our results demonstrated that the co-expression network adhered to the scale-free network principles, where the correlation coefficient for log(k) was greater than 0.85 for nodes with a degree of connection k compared with log(P(k)) representing the probability of their occurrence. Opting for β = 8 ensured network scalability. A topology matrix was crafted by transforming the expression matrix into an adjacency matrix. To cluster genes, we adopted Tom’s average linkage hierarchical clustering method. A hybrid dynamic cut tree required at least 100 genes within each gene network module. A new module emerged through clustering the modules, bringing them into closer alignment, and specifying parameters such as height = 0.15, deep Split = 2, and minimum module size = 1.

### Identification and evaluation of tumor subtypes

Survival package coxph function was used for univariate COX analysis of key genes. The NMF function of the NMF package was employed for clustering the 103 genes, resulting in the division of 431 tumor samples into two subtypes with K = 2. Immune scores for these subtypes were predicted using the estimated scores. Enrichment scores for each channel were calculated through the ssGSEA method, using the c2.cp.kegg.v7.0.symbols.gmt set as the background for the GSVA package.

### Molecular model construction and model evaluation

Differential gene expression analysis for the two subtypes employed the limma package, with false discovery rate (FDR) < 0.05 and log2|fold change|> log2(1.5) criteria for screening. Univariate COX analysis for the differentially expressed genes was performed using the survival package’s coxph function. LASSO regression analysis involved the glmnet R software package, further compressing differential genes to streamline the risk model’s gene count. We employed the GSE17536 external dataset from NCBI's GEO database, performing multivariate Cox analysis to verify the risk model’s stability by calculating coefficients for related genes. For assessing the risk score’s relationship with immunotherapy effects, we obtained TCGA immunotherapy data for CRC through TICA.

### Cell culture, RNA extraction, reverse transcription, and PCR were performed in this study

Human normal colon epithelial cells (HCoEpiC) were procured from Mingzhou Bio and cultured in 90% high-glucose Dulbecco’s modified Eagle’s medium (DMEM) supplemented with 10% fetal bovine serum (FBS; PM150210B; Procell Life Science & Technology Co., Ltd). SW620 and COLO205 cells were obtained from Procell Life Science & Technology Co., Ltd. SW620 cells were cultivated in 90% high-glucose DMEM supplemented with 10% FBS (PM150210B; Procell Life Science & Technology Co., Ltd), while COLO205 cells were cultured in RPMI-1640 supplemented with 10% FBS (PM150110B; Procell Life Science & Technology Co., Ltd.,). Subsequently, 1 × 10^5^ HCoEpiC, SW620, and COLO205 cells were seeded in a six-well plate and cultivated until reaching 90% confluency. Total RNA was extracted and followed by reverse transcription. After RNA extraction, polymerase chain reaction (PCR) detection was conducted for SLC2A3, MMP11, SCARA3, GPC1, PHGR1, OLFM2, L1CAM, CRABP2, TFF1, and CLCA1, employing the primers listed in Additional file 1: Table S1. Finally, gel electrophoresis was performed. PCR was initiated with predenaturation at 95 °C for 5 min, followed by cycles of 95 °C for 10 s (denaturation), annealing at 60 °C for 10 s, and extension at 72 °C for 20 s. This was repeated for 35 cycles, and a final extension was performed at 72 °C for 5 min.

### Statistical analysis

The statistical analyses were performed using R software 4.0.2. Data were compared between the test and control groups using the Student's t-test, and P < 0.05 indicated a significant difference.

## Results

### Colorectal cancer single-cell sequencing data were divided into 12 cell subpopulations

Utilizing NCBI’s GEO database, we obtained single-cell data from GSE178318, comprising six primary CRC samples, six liver metastasis samples, and three PBMC samples. The Seurat package facilitated the analysis of single-cell sequencing data from the six primary tumors, encompassing 25,120 genes and 55,042 cells. Additional file 1: Figure S1 depicts the outcomes of the quality control analysis.

**Fig. 1:**
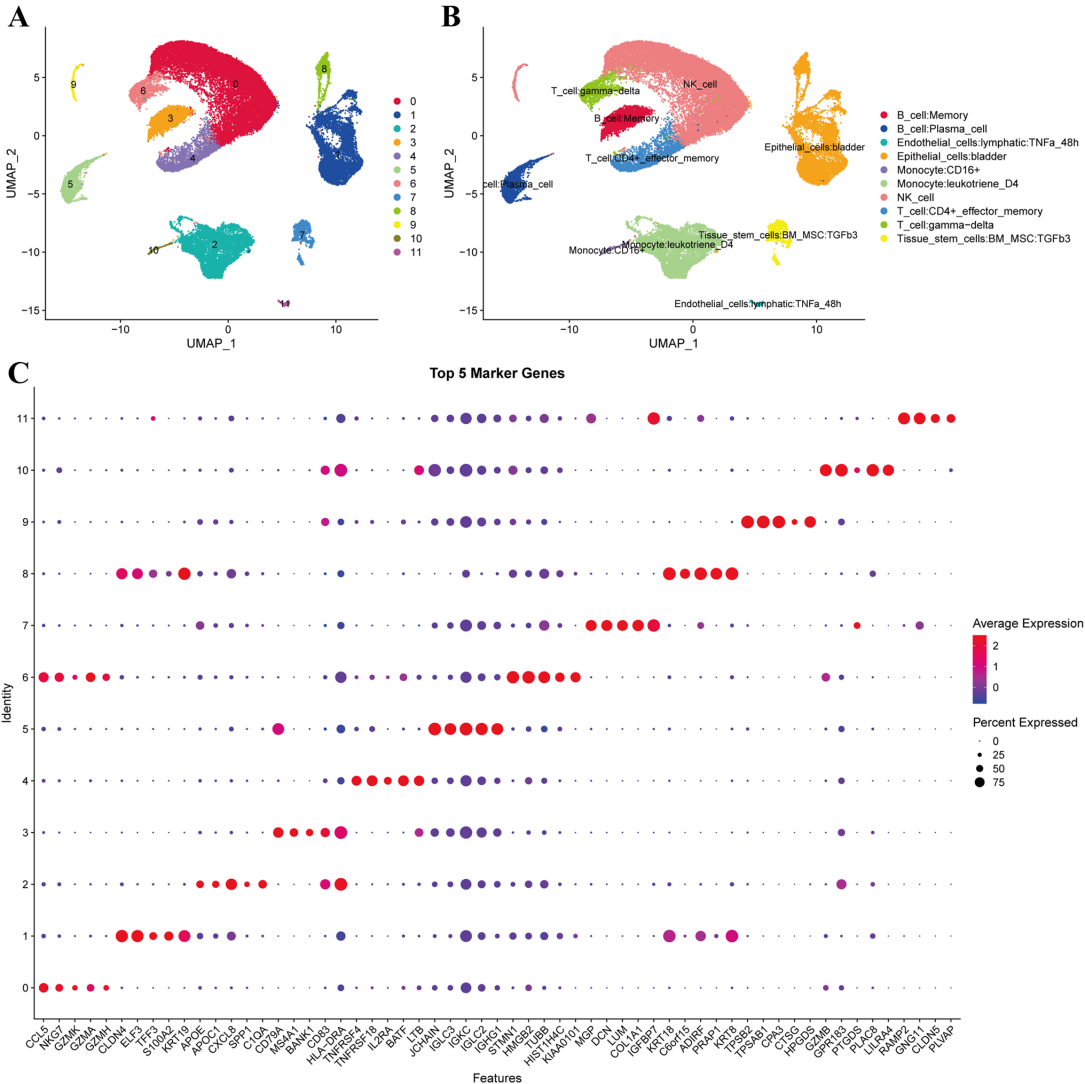
Cell clusters and top five markers of each subpopulation. **A** UMAP distribution map of 12 subgroups (each point is a cell). **B** UMAP distribution map of subgroups annotated using singleR. **C** Expression dot map of the top five marker genes of the 12 subgroups. The size of the dots represents the proportion of cells in the subgroup that express a certain gene, and the color represents the intensity of gene expression

The initial 2000 hypervariable genes were screened out, and PCA was performed using these hypervariable genes to transform high-dimensional data into low-dimensional data. Employing ElbowPlot with a 0.1 resolution on the first 30 principal components using FindAllMarkers produced a total of 12 subpopulations (Fig. 1A). Leveraging SingleR, we annotated these subgroups, resulting in the identification of 10 subpopulations (Fig. 1B). Moreover, employing the FindAllMarkers function, we pinpointed marker genes for these 12 subpopulations using a multiple of difference of 0.5, FDR < 0.05, and the smallest expression cell ratio of the subpopulation as 0.35 (Fig. 1C).

### Natural killer cell subpopulation score in TCGA-COAD was significantly different and had a prognostic value

**Fig. 2:**
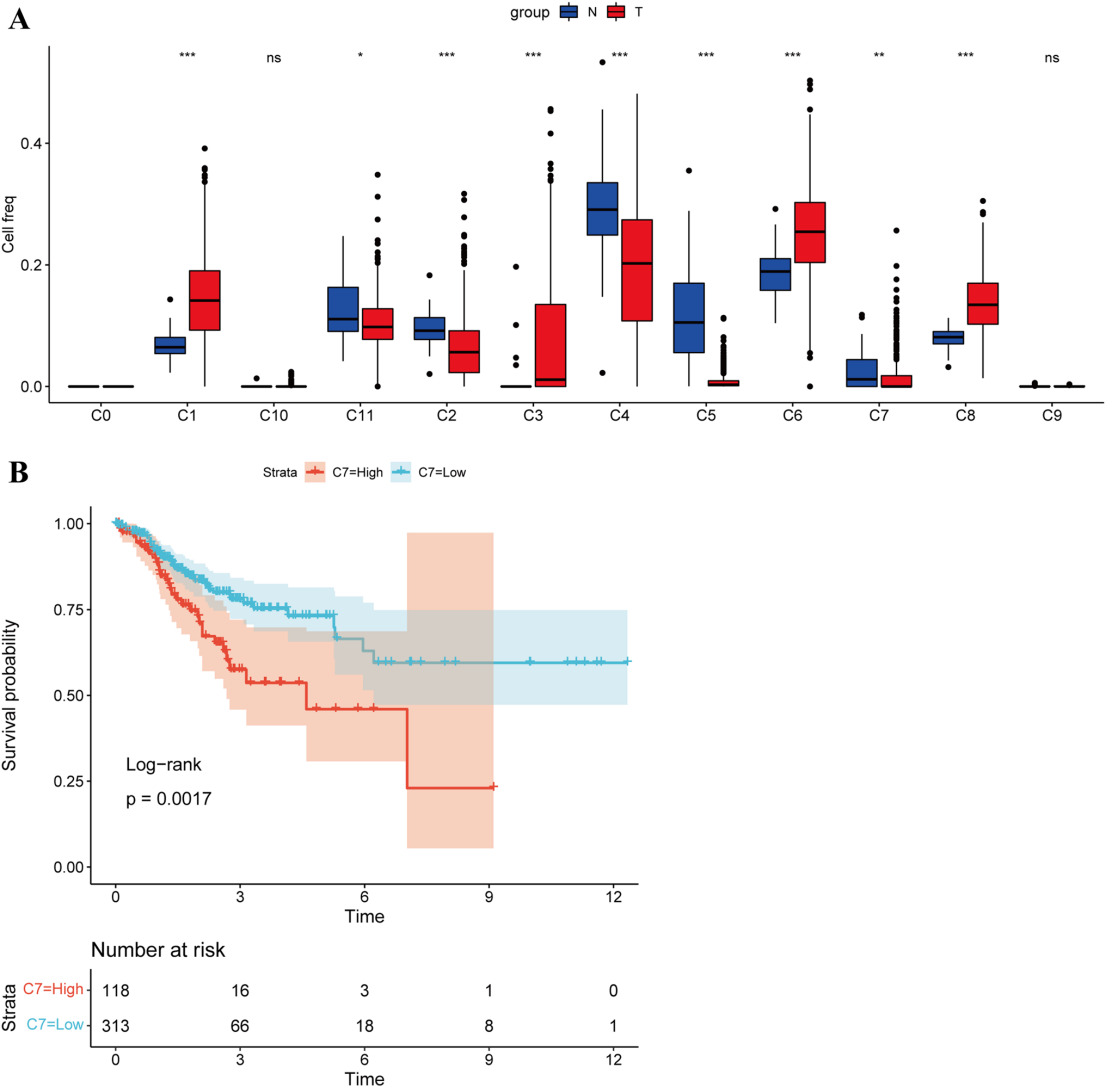
Score of each subpopulation in the TCGA dataset. **A** Cibersort function predicts the scores of the C0-C11 subgroups of each sample in the TCGA dataset; **B** influence of the high- and low-score groups of the C7 subgroup on prognosis

Data concerning FPKM and clinical information for COAD were downloaded from TCGA and subjected to filtering and logarithmic transformation. In conjunction with the marker genes identified through single-cell analysis, the CIBERSORT package’s cibersort function predicted scores for each TCGA sample across these 12 subpopulations. Through t-test analysis, we determined that the scores for nine subpopulations, excluding C0, C10, and C9, exhibited significant differences between the tumor and normal samples (Fig. 2A). Specifically, only the high- and low-score groups of the C7 subpopulation demonstrated prognostic values, after dividing them based on their average cell scores (Fig. 2B and Fig. S2).

### Screening for differentially expressed genes significantly associated with the C7 module

**Fig. 3:**
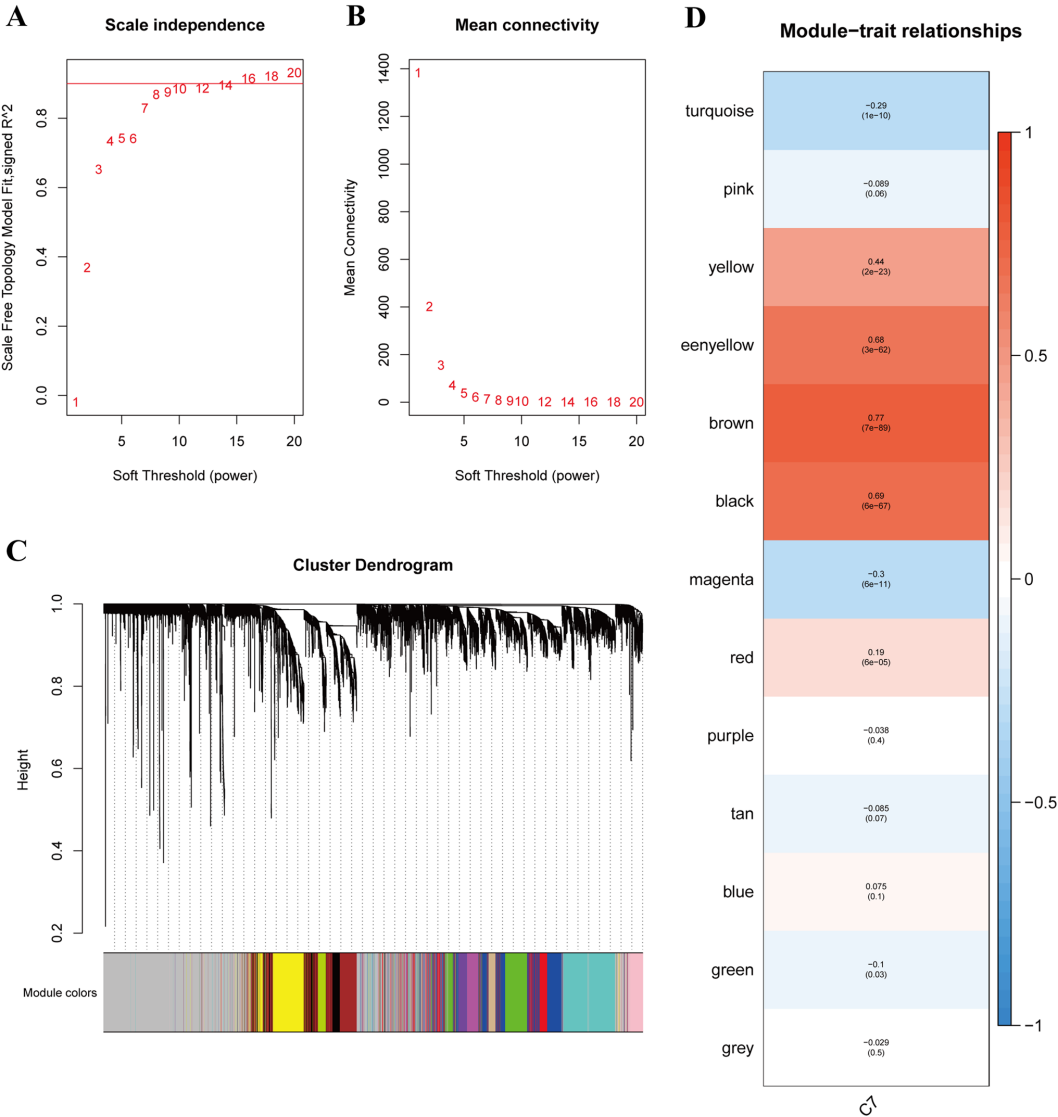
WGCNA analysis of the data in TCGA-COAD. **A** The nature of the network topology is constructed with unique power values. **B** Relationship between power values and average connectivity. **C** Genes clustered into discrete modules. **D** Correlation between each module and the C7 subgroup. The darker the color, the more significant the correlation

To mine co-expressed coding genes and co-expression modules, the WGCNA co-expression algorithm was applied to the expression profiles of coding genes from TCGA analysis. This yielded 13 modules, as depicted in Fig. 3A–C. The gray modules represented gene sets that could not be classified into other modules. Correlations between each module and the C7 subpopulation were examined, and the green-yellow, brown, and black modules demonstrated in Fig. 3D displayed the highest significant positive correlation with C7. These three modules collectively contained 1115 genes (Table S2). Subsequently, a screening process identified 202 key genes with GS > 0.6 and MM > 0.7 (Table S3).

### Clinical data typing and an immune score of natural killer cells

**Fig. 4:**
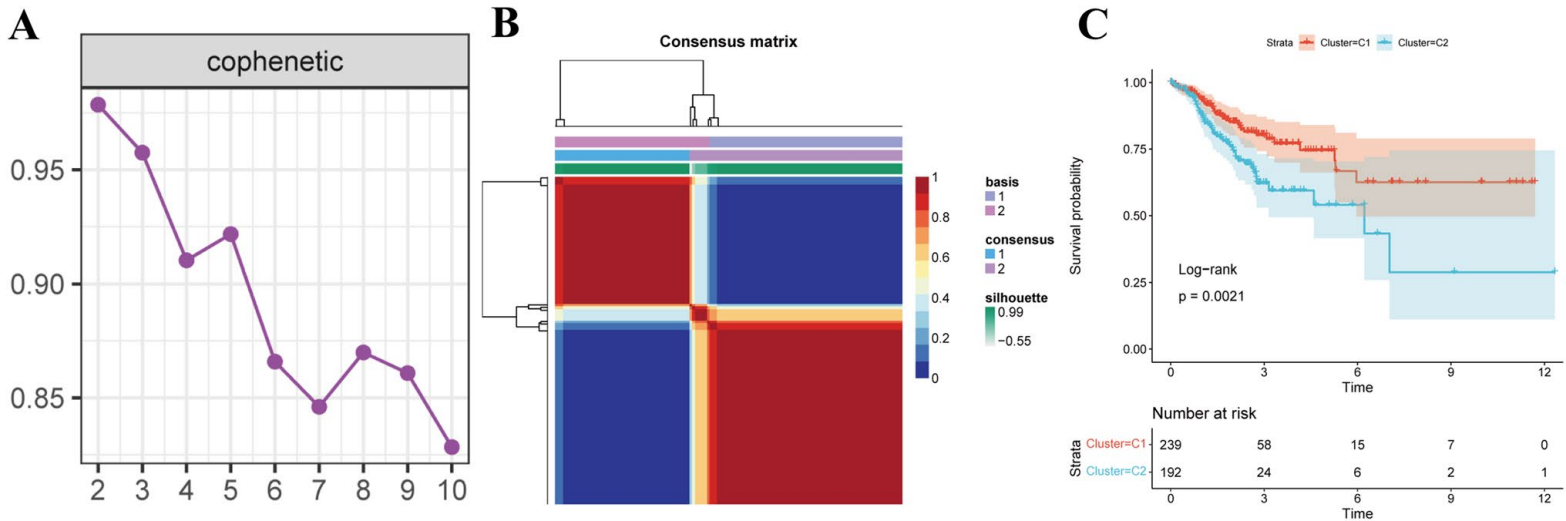
Types of TCGA-COAD samples. **A** Consensus map of NMF clustering. **B** Sample cluster of TCGA-colorectal cancer. **C** The proportion of C7 cells in two molecular subtypes of colorectal cancer

Following univariate COX analysis of the 202 key genes, we identified 103 prognostic genes with a P value of less than 0.05. Employing the NMF package’s nmf function, we clustered the 103 genes, resulting in the division of 431 tumor samples into two subtypes when k = 2 (Fig. 4A and B, Fig. S3). These subtypes displayed significant associations with prognosis (P < 0.05), with L2 indicating a poor prognosis and C1 indicating a favorable one (Fig. 4C).

**Fig. 5:**
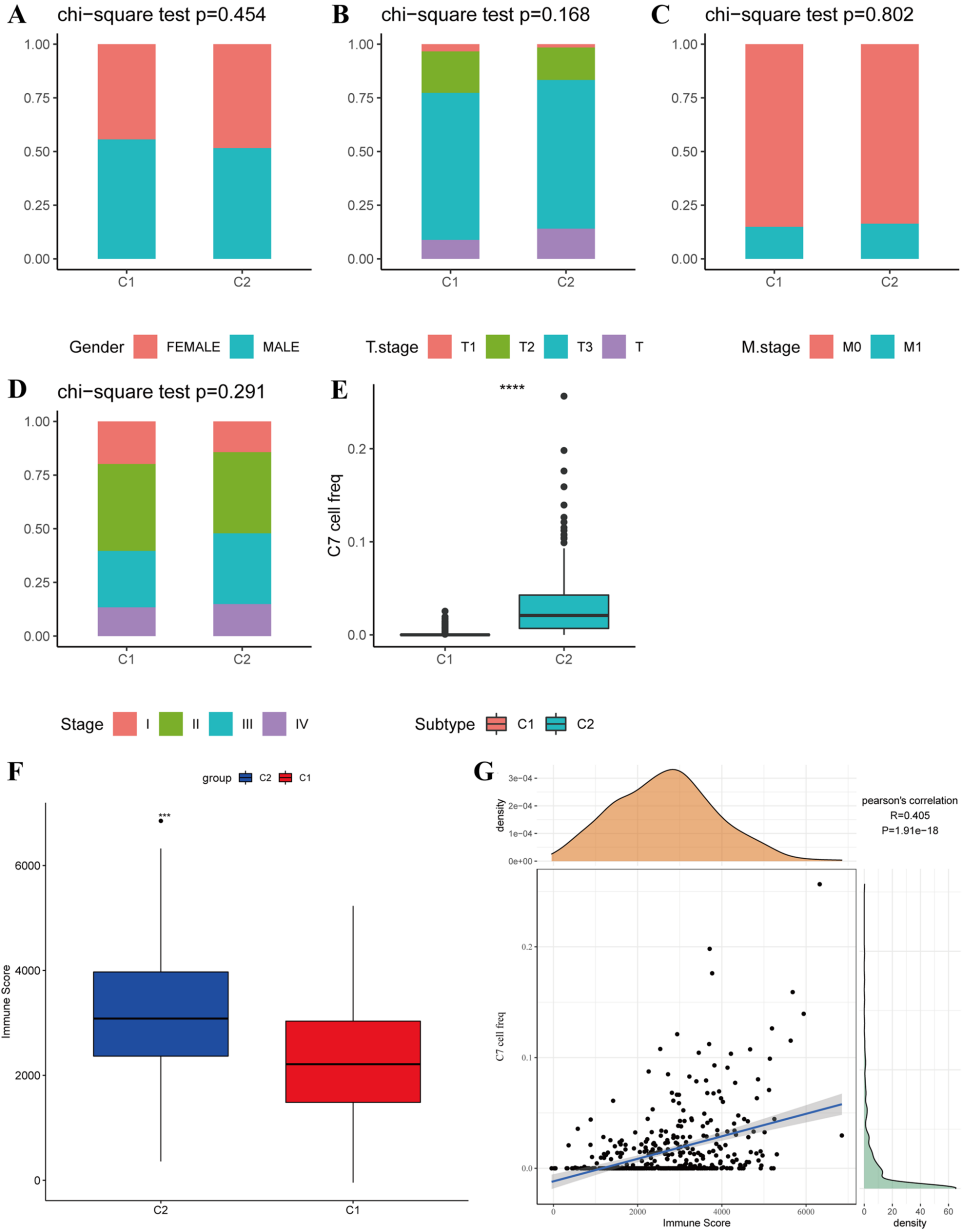
Clinical characteristics of the C1 and C2 subtypes. **A**–**D** Chi-square test results of the detection of the clinical phenotypic differences between C1 and C2 in sex and TNM stage. **E** Chi-square test results of the detection of the difference between C1 and C2 in the C7 score. **F** Estimate predicts the immune scores of C1 and C2. **G** Relationship between the immune score and the abundance of C7 in TCGA-COAD

Clinical symptom counts for these two subtypes unveiled no significant difference in sex or TNM stage between the two subgroups (Fig. 5A–E). However, subgroup scores for the two subtypes C7 subpopulation are significantly different, and the L2 subtype had a higher score than the C7 subpopulation.

Subsequent immune score predictions indicated a higher immune score for L2 than for L1 (Fig. 5F). Notably, a positive correlation was observed between the immune scores of each TCGA sample and the abundance scores of the C7 subgroup (Fig. 5G). To better comprehend these subtypes’ functions, pathway enrichment scores were calculated using ssGSEA for visualization of the top 20 pathways with the most significant differences (Fig. S4).

### Construction of clinical prognosis molecular model

**Fig. 6:**
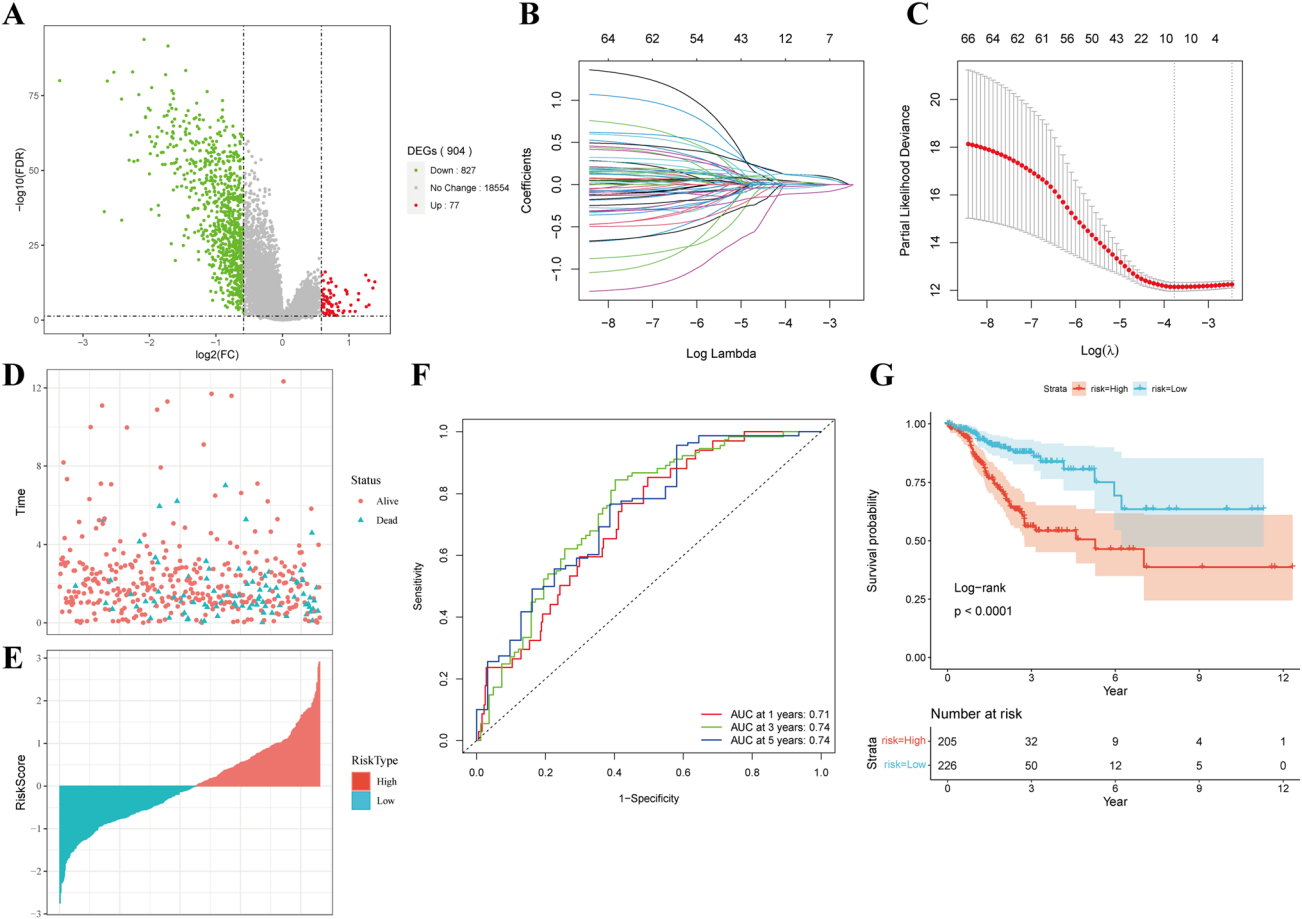
Molecular model constructed by LASSO regression. **A** Different genes between C1 and C2, where FDR < 0.05 and log2|fold change|> log2 (1.5). Use the R software package glmnet to perform LASSO COX regression analysis and analyze the change trajectory of each independent variable (**B**) to analyze the confidence interval (**C**) under each lambda. **D**, **E** Risk score distribution of each sample in the TCGA dataset. **F** Use the R software package time ROC to perform ROC analysis for the prognostic classification of the data in the TCGA-colorectal cancer dataset for 1, 3, and 5 years. **G** KM curves of risk score high-risk group and low-risk group after Z score of TCGA-colorectal cancer dataset

Based on the aforementioned clinical subtypes, we carried out differential gene expression analysis. As shown in Fig. 6A. 77 genes were upregulated while 827 genes were downregulated. Survival analysis for these genes was performed, resulting in the identification of 66 prognostically relevant genes. Given the extensive number of genes, the necessity to streamline the range while retaining high accuracy was evident.

In the pursuit of a risk model, the 66 genes were further condensed using LASSO regression. LASSO Cox regression analysis was conducted using the R software package glmnet. The optimal value for the model emerged as lambda = 0.02304054 (Fig. 6B and C). This selected lambda value would subsequently be employed to determine target genes in the subsequent step.

The final 10-gene signature formula is as follows:

$${\text{RiskScore}} = \,0.{153}*{\text{SLC2A3}} + 0.0{59}*{\text{MMP11}} + 0.{132}*{\text{SCARA3}} + 0.{148}*{\text{GPC1}} - 0.{132}*{\text{PHGR1}} + 0.0{86}*{\text{OLFM2}} + 0.0{3}*{\text{L1CAM}} + 0.0{54}*{\text{CRABP2}} - 0.0{77}*{\text{TFF1}} - 0.0{42}*{\text{CLCA1}}$$

Figure 6D and E illustrate the distribution of RiskScore scores for TCGA dataset samples, based on their expression levels. ROC analysis of RiskScore’s prognostic classification was facilitated using the timeROC package, displaying the model’s efficiency across 1, 3, and 5 years (Fig. 6F). Notably, the AUC line area was relatively substantial. Finally, Risk scores underwent z-score normalization, with a high-risk group constituted by samples with scores above zero, and a Low-risk group encompassing those with scores below zero. The Kaplan–Meier curves further validated that the significant difference is evident (P < 0.0001; Fig. 6G).

### Ten-gene signature molecular model was verified using an external dataset

**Fig. 7:**
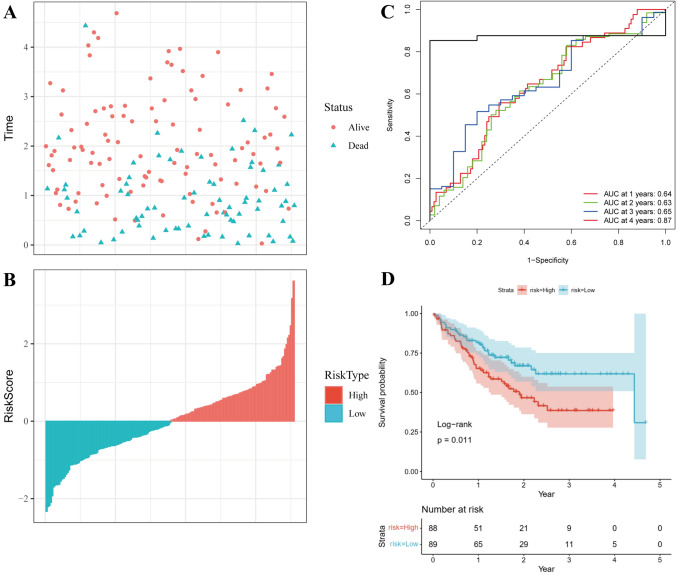
GSE17536 used for verifying the robustness of the molecular model. **A**, **B** Risk score distribution of each sample in the GSE17536 dataset. **C** Use the R software package time ROC to perform ROC analysis for prognostic classification of the data in the GSE17536 dataset for 1, 3, and 5 years. **D** KM curve of risk score high-risk group and low-risk group after Z score of GSE17536 data

**Fig. 8:**
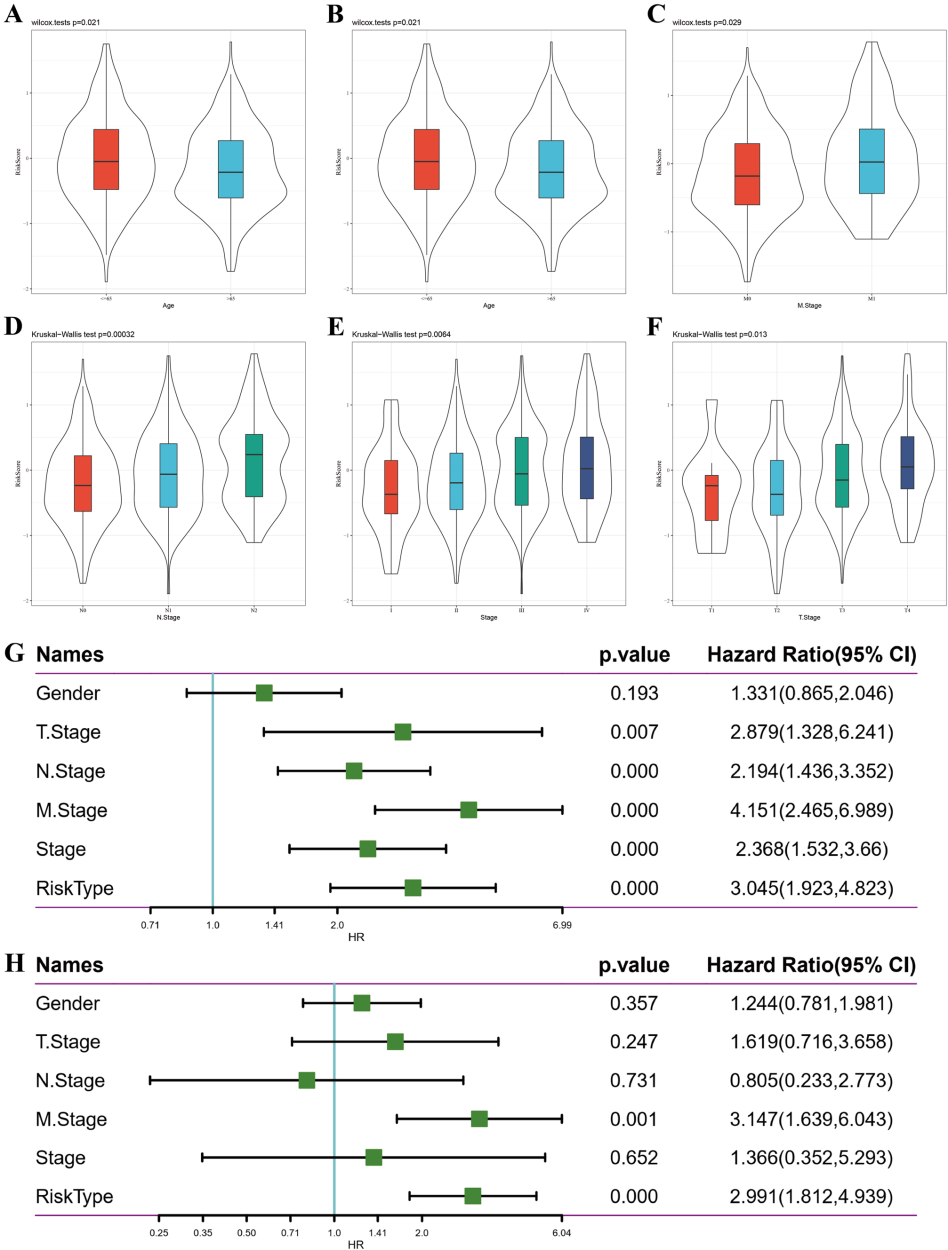
Using molecular models to analyze each sample of TCGA-COAD. Compare the risk score of different ages (**A**), sexes (**B**), and clinical stage (**C**–**F**) in the TCGA-colorectal cancer dataset. Single-factor COX (**G**), and multifactor COX (**H**), regression analysis of the relationship between risk type and various clinical features in the TCGA-colorectal cancer dataset

For validation purposes, a third dataset, GSE17536, was employed to assess the robustness of the identified 10 genes (Fig. 7). To assess the clinical applicability of the 10-gene signature model, univariate and multivariate COX regression analyses were conducted on the entire TCGA-COAD dataset to ascertain associated hazard ratio (HR), 95% confidence interval (CI) of HR, and P values. Notably, a comprehensive exploration of TCGA patient records’ clinical information, including age, sex, stage, and our RiskType information, was undertaken (Fig. 8A–F). Furthermore, univariate Cox regression analysis established a significant association between risk score and survival in the TCGA dataset (Fig. 8G). This relationship endured even in multivariate Cox regression analysis, underscoring a significant correlation between risk type (HR = 2.04, 95% CI 1.03–4.04, P = 0.05) and survival (Fig. 8H). Additionally, survival demonstrated a substantial correlation with the M stage in both univariate and multivariate analyses. Consequently, our 10-gene signature model exhibited effective predictive abilities.

**Fig. 9:**
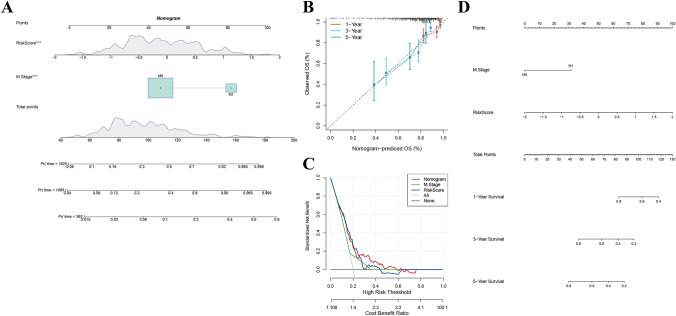
Nomogram analysis of data in TCGA-COAD. **A** The clinical features of the M stage and risk score are combined to build a nomogram model. We use the TCGA dataset to build a nomogram for the combination of the M stage and risk score. **B**–**D** Construction of the nomogram model using a combination of age, sex, T stage, N stage, and stage with a risk score

With the TCGA dataset as the basis, a nomogram was constructed by integrating M stage and risk score (Fig. 9A). Notably, the model’s performance demonstrated that the use of these 10 genes as a risk model yielded optimal survival predictions, as evidenced by the risk score feature. DCA plots for T stage, N stage, risk score, and nomogram highlighted the superior results of the nomogram (Fig. 9B–D).

**Fig. 10:**
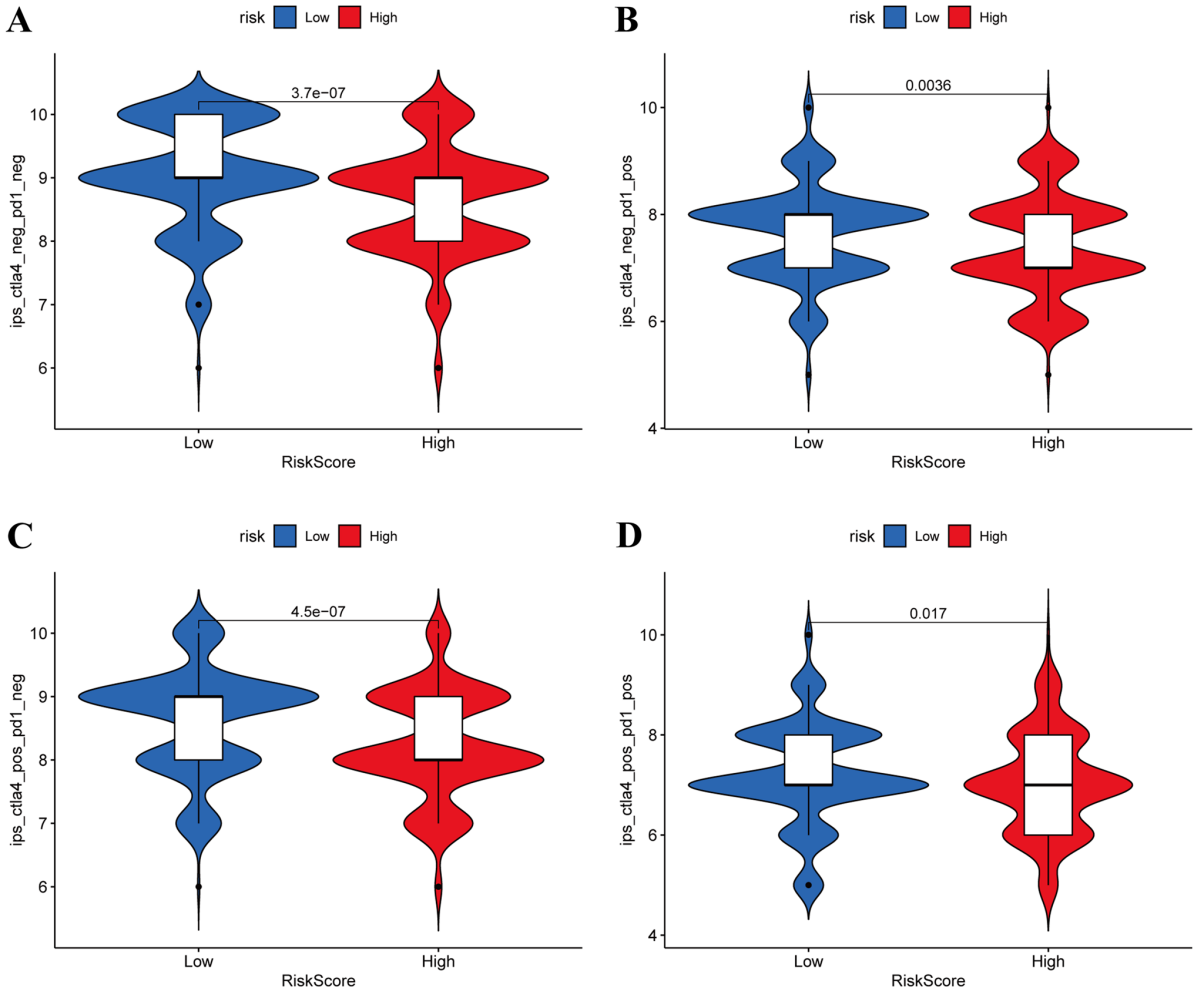
Using TCGA-colorectal cancer data on immunotherapy to analyze the effect of risk score **A**–**D**

In assessing the risk score's implications for immunotherapy effects, data concerning TCGA immunotherapy for CRC were procured through TICA. These data underscored notable differences between the low-risk and high-risk groups in terms of immunotherapy efficacy (Fig. 10A–D).

**Fig. 11:**
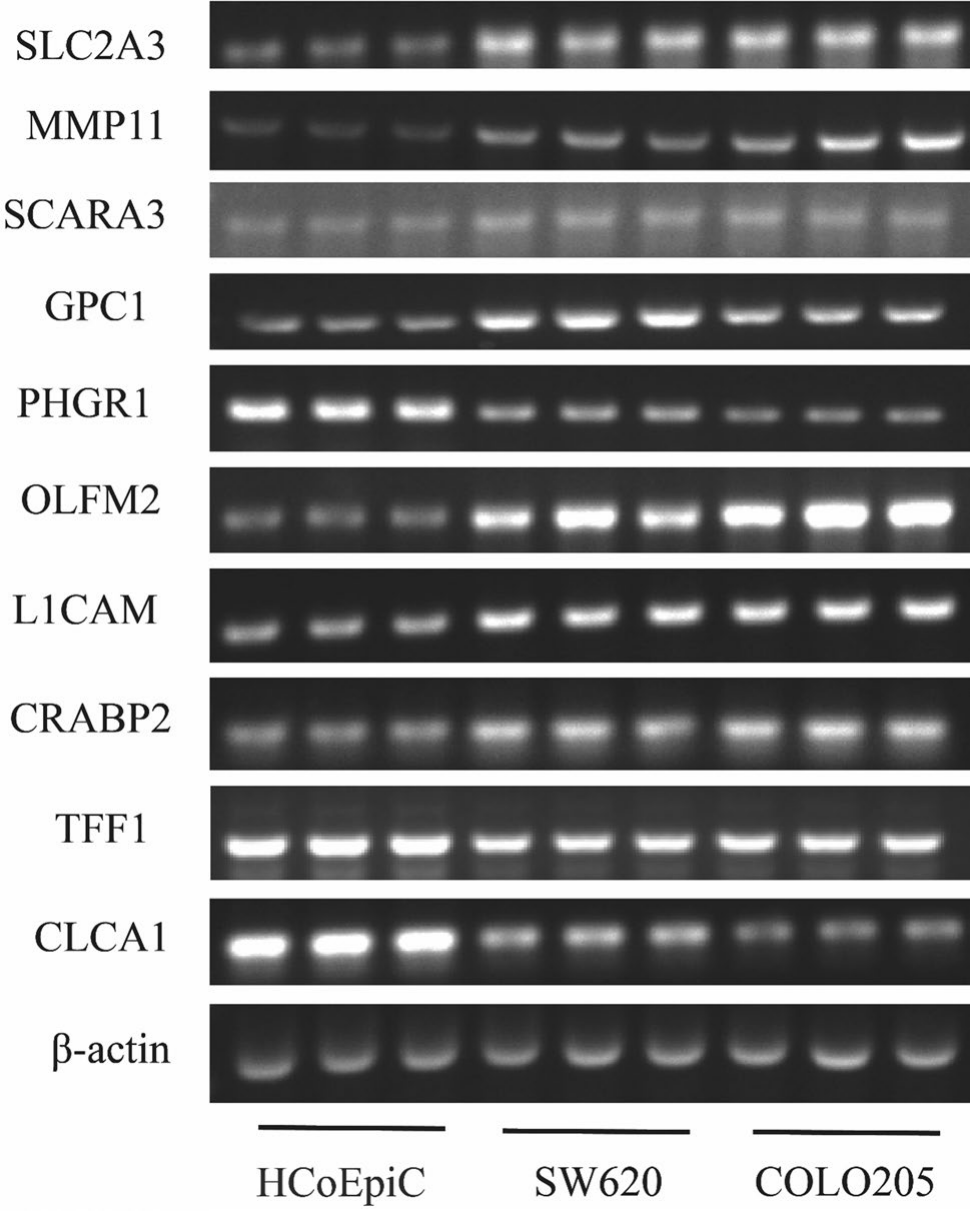
Expression of SLC2A3, MMP11, SCARA3, GPC1, PHGR1, OLFM2, L1CAM, CRABP2, TFF1, and CLCA1 in HCoEpiC, SW620, and COLO205 cells was detected by PCR. Each sample was tested with three replicates

Furthermore, we gauged the expression of SLC2A3, MMP11, SCARA3, GPC1, PHGR1, OLFM2, L1CAM, CRABP2, TFF1, and CLCA1 in HCoEpiC and compared them with SW620 and COLO205 CRC cells. Our findings indicated upregulated expression of SLC2A3, MMP11, SCARA3, GPC1, OLFM2, L1CAM, and CRABP2 in CRC cells, with the expression of PHGR1, TFF1, and CLCA1 being diminished. Importantly, these results were consistent with our molecular risk model outcomes (Fig. 11).

## Discussion

CRC is a malignant tumor that poses a great health threat. Currently, the primary method for transcriptional profiling analysis of CRC utilizes data from TCGA. Pan et al. conducted a bioinformatics-driven analysis of CRC's transcriptome and clinical data within TCGA, resulting in the development of an immune gene composition–based prognostic model [18]. Our research commenced with single-cell sequencing, progressing to the clustering of single-cell data and integration with transcriptomic and clinical data from TCGA for comprehensive analysis. This exploration unveiled a significant link between natural killer (NK) cells and the prognosis of CRC patients. NK cells play a dual role in cancer development and serve as a frontline defense against it [19–21]. Current research underscores that various cancers display dysregulated immunomodulatory signals within NK cells, undermining their monitoring and control of cancer cells [22–25]. As they are unable to maintain their immune function, dysfunctional NK cells allow some cancer cells to evade immune surveillance [25, 26].

The C7 (NK cells) subtype potentially assumes a crucial function in the genesis and progression of CRC. Research findings suggest a tight correlation between the C7 subtype and CRC’s prognosis and immune microenvironment. This connection might arise from the C7 subtype’s pivotal involvement in tumor-immune cell interactions. It could influence CRC prognosis by affecting the immune microenvironment. For instance, NK cells might sway immune cell infiltration and activation, thereby affecting the growth and spread of tumors [27]. Additionally, NK cells might influence CRC prognosis by modulating gene expression and signal transduction within tumor cells [28]. Yet, the specific role and mechanism of the C7 subtype in CRC necessitate further investigation. Subsequent studies should delve into the intricate interplay between the C7 subtype, immune microenvironment, and tumor cells, and their collective affects CRC prognosis.

Our approach encompassed WGCNA analysis of RNA-seq data and clinical information from CRC patients within TCGA database. Combining this with NK cell gene expression, we discovered correlations between the expressions of the green-yellow, brown, and black modules within NK cells. Following gene screening within these modules, CRC samples from TCGA were categorized into 2 subtypes based on the expression of associated genes. Validation revealed no distinctions in TNM, age, and sex between the L1 and L2 subtypes, but a significant discrepancy in TNM score, with the C7 subpopulation score being higher in L2. Moreover, a positive correlation existed between the immune score and the C7 subpopulation score. Notably, the two subgroups exhibited significant differences across multiple signaling pathways, including nitrogen metabolism, JAK/STAT signaling pathway, hedgehog signaling pathway, and intestinal immune network IgA production. Then, we analyzed the L1 and L2 differentially expressed genes, compressed the differentially expressed genes using LASSO regression, and finally constructed a risk index model of 10 genes. We validated the model using several data sets in a series of tests. All the results show that the model is useful in clinical situations. It was also effective in the evaluation of patients with CRC undergoing immunotherapy. Examination of the 10-gene signature molecular model's gene functions in existing literature indicated that high expression of SLC2A3 [29], MMP11 [30], SCARA3 [31], GPC1 [32], OLFM2 [33], L1CAM [34], CRABP2 [35] is linked to adverse cancer progression, and elevated expression of PHGR1 [36], TFF1 [37], and CLCA1 [38] is associated with a good prognosis of tumors.

These 10 genes are suspected to wield pivotal roles in the inception and advancement of CRC. For instance, SLC2A3, a glucose transporter, could promote the growth and spread of the tumor by intensifying glucose uptake upon overexpression [39]. MMP11, a matrix metalloproteinase, is known to degrade the extracellular matrix, potentially escalating tumor invasion and metastasis [40]. SCARA3, functioning as an oxidative stress scavenger, might relate to the antioxidant defense mechanism within tumors [41]. GPC1, a glycoprotein, could influence tumor growth and spread [42]. OLFM2, promoting nerve growth, may be tumor invasion and metastasis [43]. L1CAM, a cell adhesion molecule, might influence tumor invasion and metastasis [44]. CRABP2, an intracellular retinol-binding protein, might contribute to tumor growth and spread [45]. PHGR1, involved in ribosome biosynthesis, could potentially affect tumor growth and spread [46]. TFF1, present in gastric mucus, may be related to the growth and spread of tumors and a favorable tumor prognosis [47]. CLCA1, a chloride channel protein, might be linked to tumor growth and spread [48]. These genes likely affect CRC prognosis by influencing tumor cell growth, invasion, metastasis, antioxidant defense mechanisms, and the immune response within the tumor microenvironment. However, the specific roles, interactions, and effects of these genes on CRC prognosis necessitate further research. In summation, these 10 genes could potentially serve as vital biomarkers for CRC prognosis, offering insights into CRC development and aiding in prognosis prediction [39–48].

Among these 10 genes, some have been substantiated to play pivotal roles in CRC’s initiation and progression. Yet, the exact roles and interactions of these genes in the context of CRC remain subjects of further research. Additionally, different CRC subtypes might emerge through distinct signaling pathways. These disparities could affect disease severity and treatment response. For instance, certain CRC subtypes might exhibit increased sensitivity to specific chemotherapy drugs, while others may develop resistance to these agents [28]. In summary, both NK cells and these 10 genes appear to be central players in CRC’s initiation and progression, with diverse CRC subtypes evolving through signaling pathways. However, the precise mechanisms underlying these phenomena necessitate additional exploration.

In this study, we adopted an innovative approach, amalgamating WGCNA analysis, gene screening, and validation techniques to comprehensively investigate gene expression patterns and their clinical relevance within CRC. Our research yielded a 10-gene marker model—SLC2A3, MMP11, SCARA3, GPC1, OLFM2, L1CAM, CRABP2, PHGR1, TFF1, and CLCA1—that potentially exert significant roles in the occurrence, progression, and prognosis of CRC. Notably, this study marks the first instance where these genes have been collectively utilized to assess CRC risk and prognosis. Our only unveiled the potential roles of these genes in CRC but also introduced a novel risk index model that better predicts the survival rates of patients with CRC. Furthermore, we identified significant associations between the expression patterns of these genes and clinical features such as TNM stage and survival rate within CRC. These discoveries provide fresh insights into CRC's molecular mechanisms and present avenues for the development of novel prognostic markers and personalized treatment strategies. In summary, our research furnishes invaluable insights into gene expression patterns and their clinical relevance in CRC, offering a potentially invaluable risk index model for the evaluation and management of CRC patients. These newfound revelations and innovations lay the groundwork for further advancements in CRC research and treatment.

In this study, the amalgamation of TCGA data with single-cell sequencing data from CRC sheds light on the substantial role played by NK cells within the tumor microenvironment, thereby enriching our comprehension of CRC. First, single-cell sequencing unveils the cellular heterogeneity within tumors, and TCGA data can provide a wealth of clinical relevance. The combination of these two types of data provides a robust platform to comprehend the specific roles of biomarkers in tumor development. Second, combining single-cell sequencing and TCGA data enhances the precision of prediction models—such as those gauging patient survival rates or disease progression. However, this amalgamation presents certain limitations. For instance, the processing and integration of single-cell sequencing data and TCGA data might confront technical challenges, including data normalization and the elimination of batch effects. Furthermore, the quality and accessibility of TCGA data could potentially influence result accuracy. Moreover, the quality of single-cell sequencing data might also be constrained—sequencing depth limitations, for instance, might hinder the detection of all gene mutations.

## Conclusions

This study elucidated a 10-gene signature molecular model that can predict the prognosis of CRC. Our findings can be used to not only improve the efficacy of conventional treatment modalities but also predict the prognosis who are ready to start immunotherapy. Our findings will be critical in the initial diagnosis of patients’ clinical conditions.

### Supplementary Information

**Additional file 1: Figure S1.** A: Violin chart of nFeature_RNA, nCount_RNA, and percent.Ribo of cells before filtering; B: Violin chart of nFeature_RNA, nCount_RNA, and percent.Ribo of filtered cells; C: Statistics of cell number before and after filtering; D: The function FindVariableFeatures screens the first 2000 hypervariable genes (left) and selects the names of the first 20 hypervariable genes (right); E: principle component analysis through the first 2000 hypervariable genes; F: select the appropriate inflection point through ElbowPlot and further Dimensionality reduction. **Figure S2.** A-H: Effect of the high and low scores of C1, C2, C3, C4, C5, C6, C8, and C11 cell subpopulations on the prognosis. **Figure S3.** Classification of tumor sample subtypes when K = 2–9 is selected. **Figure S4.** Using the GSVA package and using c2.cp.kegg.v7.0.symbols.gmt as the background set, the ssGSEA method calculates the enrichment score of each sample and each pathway in the two subtypes C1 and C2. **Table S1.** Primer sequences used in polymerase chain reaction. **Table S2.** Genes (n = 1115) with the highest significant positive correlation with the C7 subpopulation. **Table S3.** 202 key Key genes (n = 202) identified with GS0.6 and MM0.7.

## Data Availability

The datasets used and/or analyzed during the current study are available within the manuscript and its supplementary information files.
