# Supplementary material for: Integrating TCGA and single-cell sequencing data for colorectal cancer: a 10-gene prognostic risk assessment model
Source: Discov Oncol. 2023 Sep 13;14:168. doi: 10.1007/s12672-023-00789-x (PMC10499771; doi:10.1007/s12672-023-00789-x)
Supplement: Supplementary file 1 — Additional file 1: Figure S1. A: Violin chart of nFeature_RNA, nCount_RNA, and percent.Ribo of cells before filtering; B: Violin chart of nFeature_RNA, nCount_RNA, and percent.Ribo of filtered cells; C: Statistics of cell number before and after filtering; D: The function FindVariableFeatures screens the first 2000 hypervariable genes (left) and selects the names of the first 20 hypervariable genes (right); E: principle component analysis through the first 2000 hypervariable genes; F: select the appropriate inflection point through ElbowPlot and further Dimensionality reduction. Figure S2. A-H: Effect of the high and low scores of C1, C2, C3, C4, C5, C6, C8, and C11 cell subpopulations on the prognosis. Figure S3. Classification of tumor sample subtypes when K = 2–9 is selected. Figure S4. Using the GSVA package and using c2.cp.kegg.v7.0.symbols.gmt as the background set, the ssGSEA method calculates the enrichment score of each sample and each pathway in the two subtypes C1 and C2. Table S1. Primer sequences used in polymerase chain reaction. Table S2. Genes (n = 1115) with the highest significant positive correlation with the C7 subpopulation. Table S3. 202 key Key genes (n = 202) identified with GS0.6 and MM0.7. [file 12672_2023_789_MOESM1_ESM.docx]

Additional Materials


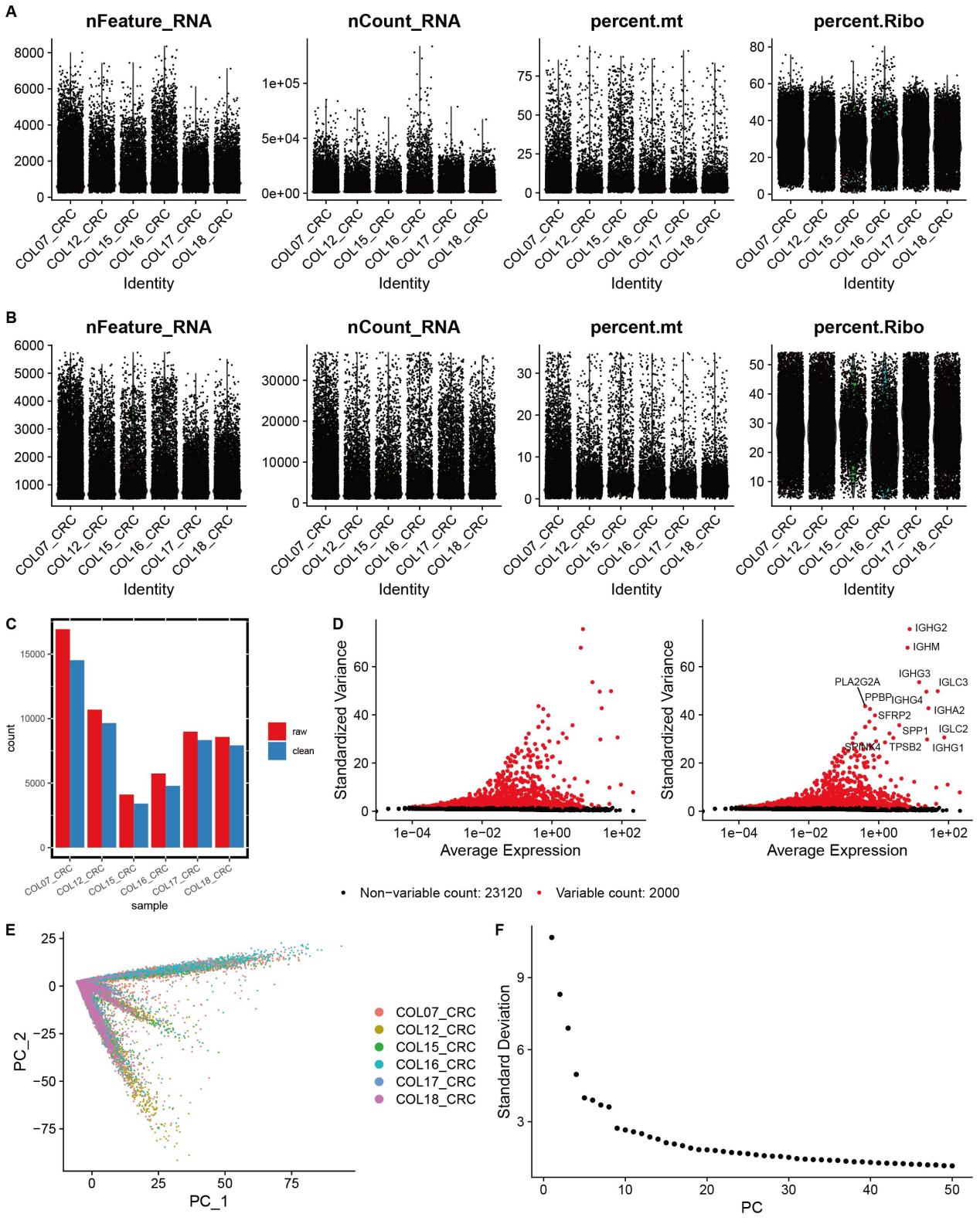


Fig. S1: A: Violin chart of nFeature_RNA, nCount_RNA, and percent.Ribo of cells before filtering; B: Violin chart of nFeature_RNA, nCount_RNA, and percent.Ribo of filtered cells; C: Statistics of cell number before and after filtering; D: The function FindVariableFeatures screens the first 2000 hypervariable genes (left) and selects the names of the first 20 hypervariable genes (right); E: principle component analysis through the first 2000 hypervariable genes; F: select the appropriate inflection point through ElbowPlot and further Dimensionality reduction.


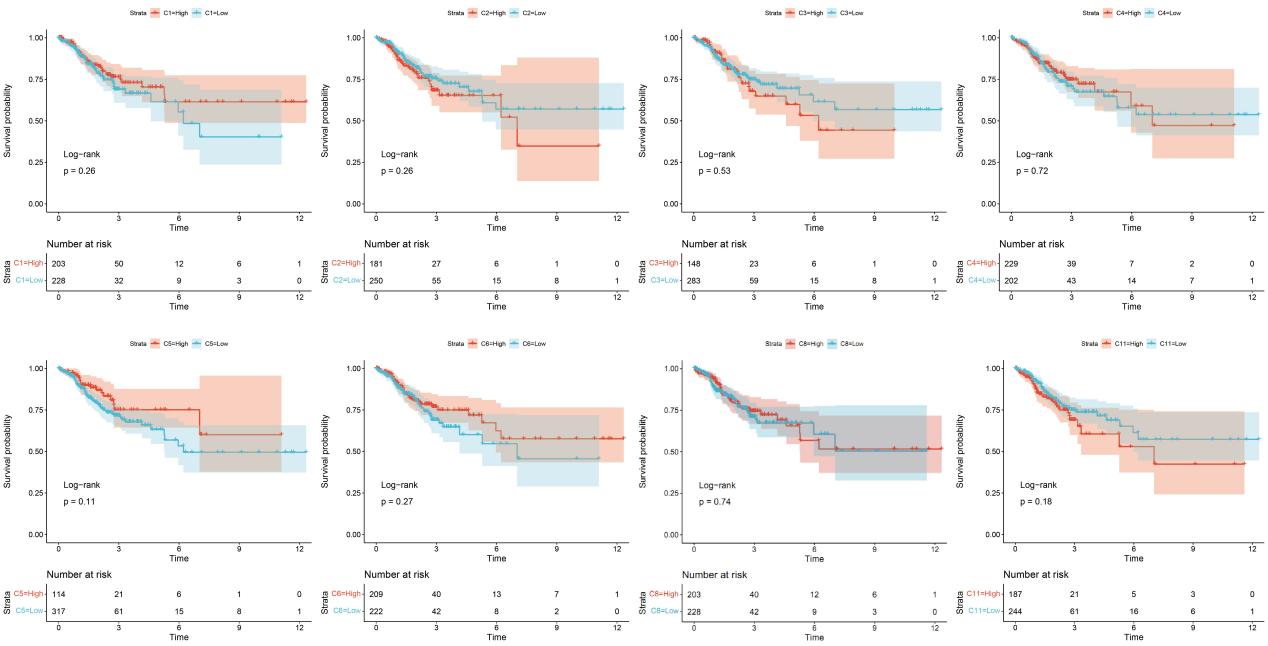


Fig. S2: A-H: Effect of the high and low scores of C1, C2, C3, C4, C5, C6, C8, and C11 cell subpopulations on the prognosis.


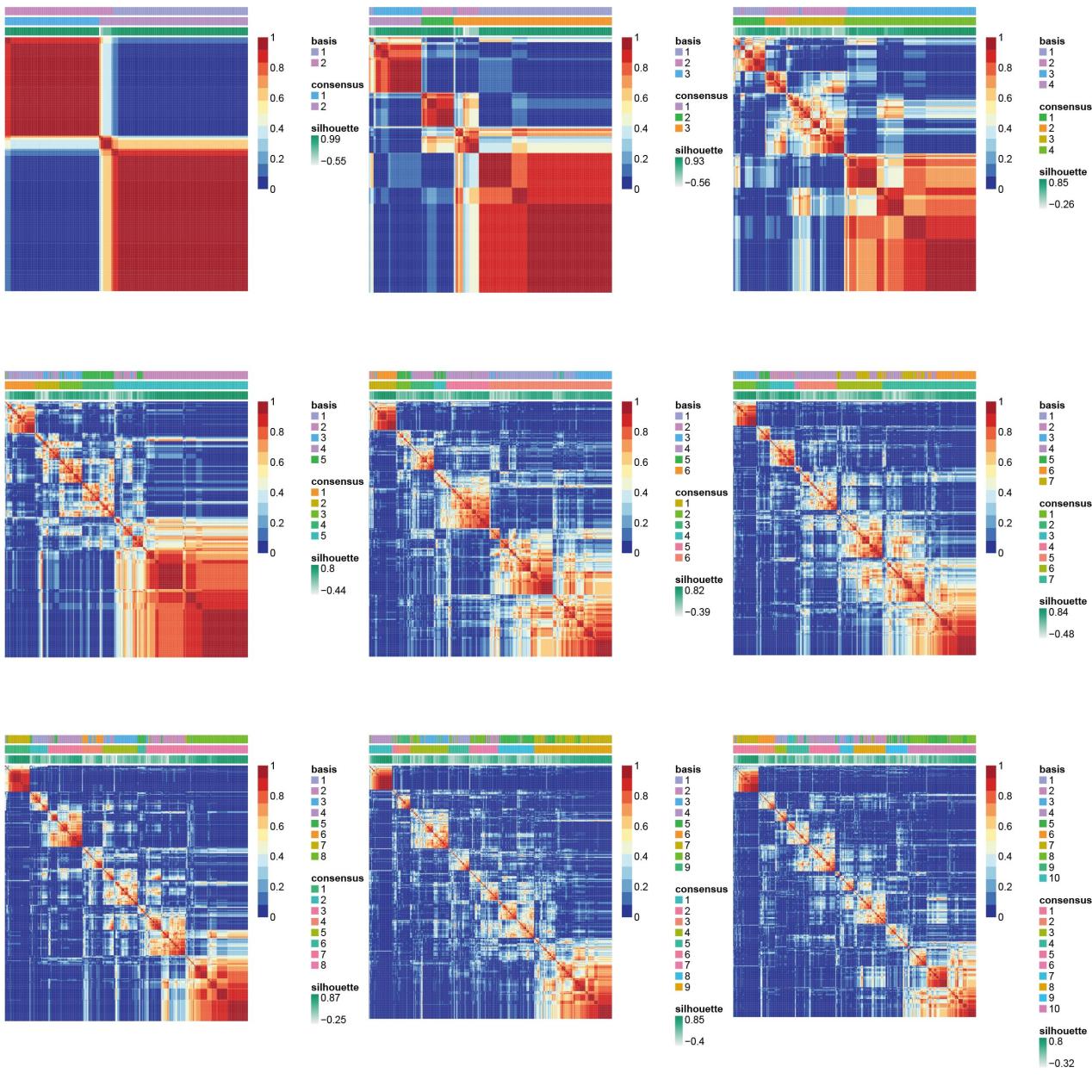


Fig. S3: Classification of tumor sample subtypes when K=2-9 is selected.


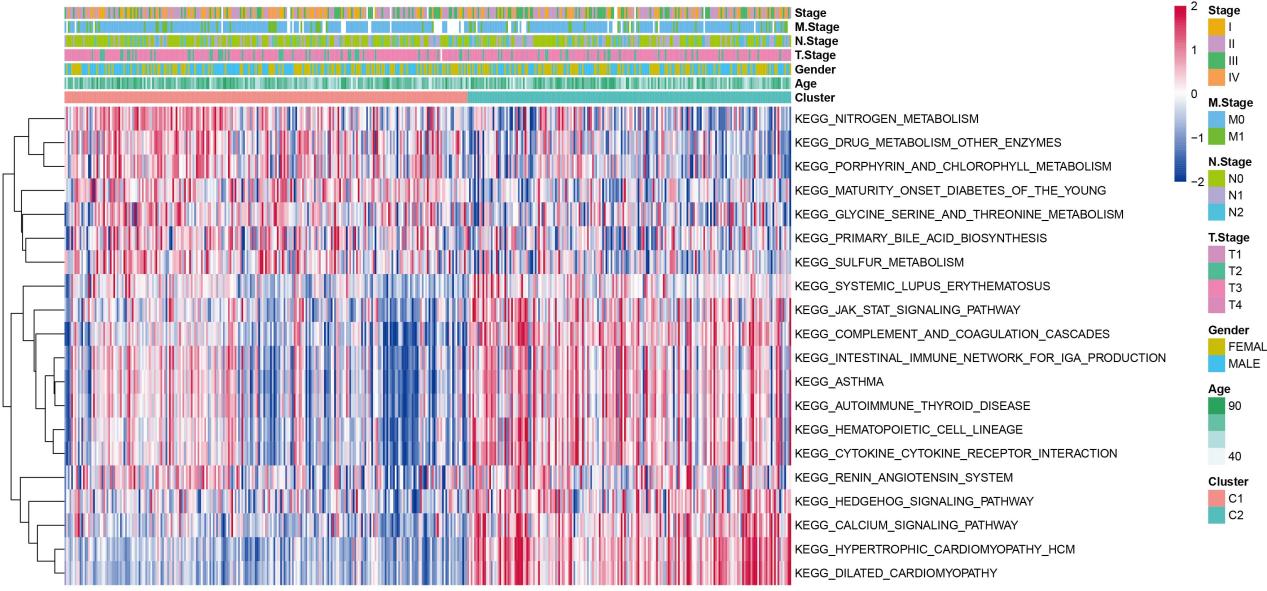


Fig. S4: Using the GSVA package and using c2.cp.kegg.v7.0.symbols.gmt as the background set, the ssGSEA method calculates the enrichment score of each sample and each pathway in the two subtypes C1 and C2.

**Table S1: Primer sequences used in polymerase chain reaction.**

| Gene | Primer | sequences |
| --- | --- | --- |
| SLC2A3 | Forward primer | CTGAGGTGCTGCTCACGTCT |
|  | Reverse primer | AGGCCAATAACCAAGCGACC |
| MMP11 | Forward primer | CTTCCCCAAGACTCACCGAG |
|  | Reverse primer | TCAGTGGGTAGCGAAAGGTG |
| SCARA3 | Forward primer | ACACATCGGTGCGGATTCTT |
|  | Reverse primer | GCAGTTGTTCAGGGCTTTCG |
| GPC1 | Forward primer | CTACGGAGCCAAGGGCTTCA |
|  | Reverse primer | GCGTGTACAGCTCTCCGAA |
| PHGR1 | Forward primer | ATGGACCCAGGTCCGAAGG |
|  | Reverse primer | TCAGTGATGTGGACCAGGGG |
| OLFM2 | Forward primer | GCGCCTCTGAGAAAATCACTCT |
|  | Reverse primer | ACTGGGAGACGTTCTGGACC |
| L1CAM | Forward primer | CAGGCACCAGGACCATCATT |
|  | Reverse primer | TGCAGGGTCTTGTTGTGGTT |
| CRABP2 | Forward primer | GGTTGGGGAGGAGTTTGAGG |
|  | Reverse primer | TGGTCAGGATCAGTTCCCCA |
| TFF1 | Forward primer | GAGACGTGTACAGTGGCCC |
|  | Reverse primer | CTGGAGGGACGTCGATGGTA |
| CLCA1 | Forward primer | TCTCCGAAGCACATGGGAAG |
|  | Reverse primer | GTCGATTGAGGCGGTTACCA |
| β-actin | Forward primer | GGATTCCTATGTGGGCGACGA |
|  | Reverse primer | GCGTACAGGGATAGCACAGC |

**Table S2: Genes (n=1115) with the highest significant positive correlation with the C7 subpopulation.**

A2M，A4GALT，ABCA8，ABHD14B，ABHD4，ABHD8，ABI3BP，ABLIM3，ACAN，ACKR1，ACKR3，ACSL1，ACTA2，ACTG2，ACTN1，ACY1，ADAM12，ADAM19，ADAM33，ADAMTS1，ADAMTS12，ADAMTS14，ADAMTS2，ADAMTS4，ADAMTS9，ADAMTSL4，ADCY5，ADCY9，ADGRA2，ADGRF5，ADGRL2，ADGRL4，ADH1B，ADIPOQ，AEBP1，AHDC1，AHNAK2，AKAP12，AKT3，ALDH1A3，ALDH1L2，ALPL，AMIGO2，AMOTL1，AMOTL2，ANGPTL1，ANGPTL2，ANGPTL4，ANKRD65，ANOS1，ANTXR1，ANXA1，ANXA5，ANXA6，AOC3，AOX1，APBB1，APLNR，APOD，AQP1，ARAP1，ARHGAP23，ARHGAP31，ARHGEF17，ARHGEF25，ARL4C，ARL4D，ARMCX1，ARMCX2，ARNT2，ARSI，ASB2，ASPN，ATP1A2，ATP2B4，ATP5MC3，ATP8B2，AXL，B3GALNT1，B3GNT9，BACE1，BAIAP2L2，BASP1，BCAM，BCAT1，BCHE，BCL2L11，BCL6，BCL6B，BCL9L，BDH1，BEX1，BEX4，BGN，BHLHE41，BICC1，BMERB1，BMF，BMP1，BMP3，BMP6，BOC，BST1，C11orf96，C14orf132，C16orf89，C1QTNF1，C1QTNF3，C1QTNF6，C1R，C1S，C20orf194，C3，C3orf80，C4A，C4B，C5orf46，C6orf136，C7，C8orf88，CA11，CACNA1C，CACNA1H，CACNA2D1，CADM1，CADM3，CALB2，CALCRL，CALD1，CALHM2，CALU，CAP2，CARD6，CARTPT，CASQ2，CAV1，CAV2，CAVIN1，CAVIN2，CAVIN3，CBLC，CBX6，CBX7，CC2D2A，CCDC102A，CCDC3，CCDC71L，CCDC8，CCDC80，CCL11，CCL21，CCN1，CCN2，CCN4，CCN5，CCNO，CD109，CD248，CD276，CD34，CD36，CD59，CD82，CD93，CD99L2，CDC42EP3，CDH11，CDH5，CDIP1，CDK14，CDKN2B，CDR2L，CDX1，CENPV，CERCAM，CFH，CFL2，CGNL1，CH25H，CHD3，CHN1，CHRDL1，CHRDL2，CHRM2，CHRNA3，CHST14，CHST15，CHST3，CHSY1，CILP，CILP2，CKMT1A，CKMT1B，CLDN5，CLEC11A，CLEC14A，CLEC3B，CLEC5A，CLIC4，CLIC6，CLIP3，CLMP，CLSTN3，CLU，CMA1，CMTM3，CMTM8，CNN1，CNN3，CNRIP1，CNTNAP1，COL10A1，COL11A1，COL12A1，COL14A1，COL15A1，COL16A1，COL18A1，COL1A1，COL1A2，COL3A1，COL4A1，COL4A2，COL5A1，COL5A2，COL5A3，COL6A1，COL6A2，COL6A3，COL7A1，COL8A1，COL8A2，COLEC12，COMP，COPZ2，COX5A，COX7A1，CPED1，CPM，CPNE8，CPQ，CPXM1，CPXM2，CRABP2，CRACR2B，CRIP2，CRISPLD1，CRISPLD2，CRYAB，CSGALNACT1，CSGALNACT2，CSPG4，CSRP1，CST2，CTF1，CTHRC1，CTSF，CTSG，CTSK，CTSO，CTSZ，CXCL12，CXCL6，CYB5R3，CYBRD1，CYGB，CYP1B1，CYS1，CYTH3，CYYR1，DAAM2，DACT1，DACT3，DBN1，DCBLD2，DCHS1，DCN，DDR2，DEGS1，DENND5A，DEPP1，DES，DIO2，DIP2C，DIXDC1，DKK2，DKK3，DLC1，DMD，DNAJB4，DNAJB5，DNM1，DOCK11，DPT，DPYSL3，DSE，DTX3，DYSF，EBF4，ECM1，ECM2，ECRG4，ECSCR，EDIL3，EDNRA，EDNRB，EFEMP1，EFEMP2，EFHD1，EFS，EGFL6，EGFL7，EGR2，EGR3，EHD2，EHD3，EID1，ELK3，ELMO1，ELN，EMCN，EMILIN1，EML1，EMP1，EMP3，ENG，ENTPD1，EPAS1，EPHA7，EPS8L3，EPYC，ESAM，ETS1，ETV1，EVA1B，EVC，F2R，F2RL2，F2RL3，FAAH，FABP4，FADS1，FAM107A，FAM162A，FAM167B，FAM20C，FAP，FAXDC2，FBLN1，FBLN2，FBLN5，FBN1，FBXL7，FBXO17，FBXO32，FERMT2，FES，FGD5，FGF2，FGF7，FGFR1，FHL1，FHL3，FIBIN，FILIP1L，FKBP10，FKBP7，FLNA，FLNC，FLT1，FMNL3，FMOD，FN1，FNBP1，FNDC1，FNDC4，FOSL2，FOXF1，FOXF2，FOXO1，FOXS1，FRMD6，FRZB，FSCN1，FST，FSTL1，FSTL3，FXYD6，FYN，FZD1，FZD2，FZD4，FZD8，G0S2，GABARAPL1，GADD45B，GAL3ST4，GALE，GALNT15，GAMT，GAP43，GAS1，GAS7，GASK1B，GBGT1，GCAT，GEM，GFPT2，GFRA1，GFRA3，GGT5，GJA1，GJA4，GJA5，GJB2，GLB1L2，GLI3，GLIPR1，GLIS2，GLT8D2，GNAI1，GNAI2，GNAO1，GNB4，GNG11，GNG7，GNG8，GPC1，GPC6，GPM6A，GPM6B，GPR176，GPR4，GPR68，GPRC5B，GPSM1，GPX2，GPX3，GPX7，GPX8，GRASP，GREM1，GREM2，GRK5，GRP，GSN，GSTK1，GUCY1A1，GUCY1B1，GULP1，GXYLT2，GYPC，HADH，HAND1，HAND2，HAS2，HDGFL3，HEG1，HERC5，HEYL，HIGD2A，HIP1，HMCN2，HOMER3，HOPX，HPGDS，HSD11B2，HSD17B14，HSD17B6，HSPA12B，HSPB6，HSPB7，HSPB8，HSPG2，HTR2B，HTRA1，HTRA3，IBSP，ID1，IGFBP3，IGFBP4，IGFBP5，IGFBP6，IGFBP7，IKBIP，IL1R1，IL3RA，IL6，ILK，INHBA，INMT，IQSEC1，IRS1，ISLR，ISM1，ITGA1，ITGA11，ITGA5，ITGA7，ITGA9，ITGB1，ITGB5，ITGBL1，ITIH5，ITPR1，ITPRIP，ITPRIPL2，JAM2，JAM3，JAZF1，JCAD，JPH2，JPT1，KANK2，KCND3，KCNE4，KCNJ8，KCNMA1，KCNMB1，KCTD11，KCTD12，KCTD15，KDR，KIF26B，KIF3C，KIFC3，KIRREL1，KIT，KLF11，KLF12，KLF2，KLF7，KLF9，KLHDC8B，KLHL5，L1CAM，LAD1，LAMA2，LAMA4，LAMB1，LAMB2，LAMC1，LAMP5，LARP6，LATS2，LAYN，LBH，LDB3，LDOC1，LEF1，LGALS1，LGALS4，LGI2，LHFPL6，LIMS2，LIX1L，LMCD1，LMO2，LMO3，LMOD1，LOX，LOXL1，LOXL2，LPAR1，LRP1，LRRC15，LRRC32，LRRC8A，LRRN2，LRRN4CL，LSAMP，LTBP1，LTBP2，LTBP3，LUM，LXN，LYNX1，LYVE1，LZTS1，MAB21L2，MAF，MAGEH1，MAMDC2，MAN2B1，MAOB，MAP1A，MAP1B，MAP4K4，MAP7D1，MAP7D3，MAPRE2，2-Mar，MARVELD1，MASP1，MATN3，MCAM，MDFIC，MEDAG，MEF2C，MEF2D，MEGF6，MEIS1，MEIS3，MEOX1，MEOX2，MERTK，MFAP2，MFAP4，MFAP5，MFGE8，MGP，MICAL2，MID2，MITF，MMP11，MMP13，MMP14，MMP19，MMP2，MMRN1，MMRN2，MN1，MORN5，MOXD1，MRAS，MRC2，MRGPRF，MRVI1，MSC，MSN，MSRB3，MX2，MXRA5，MXRA7，MXRA8，MYADM，MYCT1，MYH10，MYH11，MYH9，MYL9，MYLK，MYOC，MYOCD，MYOF，MYOM1，NAB2，NACC2，NATD1，NAV1，NCS1，NDN，NDST1，NES，NEXN，NFATC4，NFIC，NFKB2，NGFR，NIBAN1，NID1，NID2，NKX2-3，NLGN2，NNAT，NNMT，NOP16，NOS3，NOTCH2，NOTCH3，NOXA1，NPR1，NPTX1，NPTXR，NR2F2，NR3C1，NR4A3，NREP，NRP1，NRP2，NRSN2，NTM，NTN1，NUAK1，NXN，NXPH3，OBSL1，OGN，OLFM1，OLFM2，OLFML1，OLFML2A，OLFML2B，OLFML3，OLR1，OMD，OR51E2，ORAI2，OSMR，OVOL2，P2RY14，P3H1，P3H3，P4HA3，PACS1，PALLD，PALM，PAM，PAMR1，PAPLN，PBX1，PBX3，PBXIP1，PCBD1，PCDH18，PCDH7，PCDHGC3，PCED1B，PCOLCE，PCOLCE2，PCP4L1，PCSK5，PDE2A，PDE3A，PDE5A，PDGFB，PDGFC，PDGFD，PDGFRA，PDGFRB，PDGFRL，PDK4，PDLIM3，PDLIM4，PDLIM7，PDPN，PDSS1，PDZRN3，PDZRN4，PEA15，PECAM1，PELI2，PER1，PFKFB3，PGF，PGM5，PHLDA3，PHLDB2，PHYHD1，PI15，PI16，PIK3IP1，PIM1，PKD1，PKD2，PKIG，PLA1A，PLAC9，PLAT，PLAU，PLBD2，PLD3，PLEKHA4，PLEKHG2，PLEKHO1，PLIN1，PLIN4，PLN，PLOD1，PLP1，PLPP1，PLPP3，PLPP4，PLPPR2，PLSCR4，PLVAP，PLXDC2，PLXND1，PMP22，PNCK，PNMA1，PODN，PODNL1，PODXL，POGLUT2，POPDC2，POSTN，PPP1R12B，PPP1R14A，PPP1R18，PPP1R1A，PPP1R1B，PPP1R3C，PRAF2，PRCP，PRELP，PRIMA1，PRKD1，PRKG1，PRNP，PRR15L，PRR16，PRRX1，PRRX2，PSD，PTGIS，PTGS1，PTN，PTPRM，PTPRS，PTPRU，PXDC1，PXDN，PYGM，QKI，RAB23，RAB31，RAB34，RAB3IL1，RAI14，RAI2，RAMP1，RAMP2，RAMP3，RARRES2，RASA3，RASIP1，RASL12，RASSF8，RBMS1，RBP7，RBPMS，RBPMS2，RCAN1，RCAN2，RCCD1，RCN3，RDX，REEP2，RELB，RENBP，RERG，RERGL，RETN，RFLNB，RFTN1，RGCC，RGL1，RGMA，RGS16，RGS2，RGS4，RGS5，RHOJ，RHOQ，RIN2，RIPOR1，RNF122，RNF144A，RNF150，RNF152，RNF24，ROBO1，ROBO4，ROR2，RPS6KA2，RRAD，RRAGD，RSPO3，RTL8B，RTL8C，RUNX1，RUNX2，RUSC2，S1PR1，S1PR3，SAMD11，SAMD4A，SCARA3，SCARF2，SCG2，SCN7A，SCUBE2，SCUBE3，SDC2，SDC3，SELE，SELENOM，SELP，SEMA3G，SEMA4C，SEMA6B，SEMA7A，SERPINE1，SERPINF1，SERPING1，SERPINH1，SFRP1，SFRP2，SFRP4，SFXN3，SFXN4，SGCA，SGCD，SGCE，SH2D3C，SH3PXD2A，SH3PXD2B，SH3RF3，SHANK3，SHISA2，SHISA3，SHISA4，SHISAL1，SIPA1，SIRPA，SLC12A4，SLC16A2，SLC22A17，SLC24A3，SLC26A6，SLC2A3，SLC2A4，SLC36A1，SLC41A1，SLC43A3，SLC7A2，SLCO2A1，SLFN11，SLIT2，SLIT3，SMAGP，SMARCA1，SMIM10，SMO，SMTN，SNAI1，SNAI2，SNCG，SNN，SORBS1，SOX18，SPARC，SPARCL1，SPART，SPC24，SPEG，SPHK1，SPOCK1，SPON1，SPON2，SPP1，SPSB1，SRPX，SSC5D，SSPN，ST3GAL1，ST6GALNAC5，STARD13，STC1，STEAP4，STMN2，STOM，STON1，STUM，STX2，SUCLG1，SUGCT，SULF1，SULF2，SUN2，SUSD2，SUSD6，SVEP1，SVIL，SYDE1，SYNC，SYNDIG1，SYNM，SYNPO，SYNPO2，SYT11，TACR2，TAFA5，TAGLN，TBC1D1，TBC1D9，TCEA2，TCEAL2，TCEAL3，TCEAL7，TCF21，TCF4，TDO2，TDRP，TEK，TENT5B，TFPI，TGFB1，TGFB1I1，TGFB3，TGFBR1，TGM2，THBD，THBS1，THBS2，THBS3，THBS4，THY1，TIE1，TIMP1，TIMP2，TIMP3，TLN1，TMEM100，TMEM119，TMEM132A，TMEM158，TMEM200B，TMEM204，TMEM35A，TMEM45A，TMEM47，TMEM88，TMOD1，TNC，TNFAIP6，TNFAIP8L3，TNFRSF4，TNFSF11，TNFSF12，TNFSF4，TNS1，TNS2，TNS3，TNXB，TP53INP1，TP53INP2，TPBG，TPM1，TPM2，TPP1，TPST1，TRAM2，TRARG1，TSC22D3，TSHZ3，TSPAN11，TSPAN18，TSPAN2，TSPAN4，TSPAN9，TSPYL2，TSPYL4，TSPYL5，TTC38，TUBA1A，TUBB6，TUSC3，TWIST1，TWIST2，TWSG1，TXNIP，UBE2E2，UBTD1，UCHL1，UNC5B，USH1C，USP11，UST，VAMP2，VASH1，VASN，VAT1，VCAM1，VCAN，VCL，VEGFC，VGLL3，VIM，VIP，VPS35L，VSTM4，VWF，WASF3，WDR81，WFDC1，WNT2，WNT5A，WNT9A，WWC2，WWC3，WWTR1，XYLT1，ZBTB4，ZBTB47，ZCCHC24，ZEB1，ZEB2，ZFP36L1，ZMIZ1，ZNF134，ZNF264，ZNF304，ZNF331，ZNF362，ZNF467，ZNF469，ZNF511，ZNF532，ZNF618，ZNF853，ZNF880，ZSCAN18，ZSWIM4

**Table S3: 202 key Key genes (n=202) identified with GS0.6 and MM0.7.**

ACTA2，ADAM12，ADAMTS12，ADGRA2，AEBP1，AMOTL1，ANGPTL2，ANTXR1，AOC3，ARHGEF17，ARHGEF25，ARSI，ATP8B2，BASP1，BCL6B，BGN，BOC，C14orf132，C1R，C1S，C3orf80，CALD1，CAVIN1，CCDC8，CCDC80，CCN2，CCN4，CD34，CD93，CDH11，CERCAM，CFH，CLEC14A，CLIP3，CLMP，CMTM3，CNRIP1，CNTNAP1，COL11A1，COL14A1，COL15A1，COL1A1，COL1A2，COL3A1，COL5A1，COL5A2，COL6A3，COL8A1，COL8A2，COLEC12，COMP，COPZ2，CPXM1，CRISPLD2，CRYAB，CTHRC1，CYS1，DAAM2，DACT3，DCN，DDR2，DNAJB5，DPYSL3，DSE，EFEMP1，EFEMP2，EFS，EHD2，EMILIN1，EVC，FBLN1，FBLN2，FBLN5，FBN1，FBXL7，FERMT2，FGFR1，FIBIN，FNDC1，FNDC4，FRMD6，FSTL1，FXYD6，FZD1，GAS1，GAS7，GFPT2，GGT5，GJA5，GLI3，GLIS2，GLT8D2，GPR176，GPR68，GRASP，GYPC，HEG1，HSPA12B，HSPB8，HTRA1，IL1R1，ITGA11，ITGA5，JAM3，KANK2，KCNE4，KIRREL1，LAMA4，LAYN，LDOC1，LHFPL6，LMOD1，LTBP2，LZTS1，MAF，MAP1A，MEDAG，MFAP5，MGP，MMP14，MMP19，MMP2，MMRN2，MN1，MOXD1，MRAS，MRC2，MRGPRF，MRVI1，MSC，MSRB3，MXRA8，MYL9，NID2，NNMT，NPR1，NRP1，NRP2，NTM，NUAK1，NXN，OLFML1，OLFML2B，OSMR，P4HA3，PALM，PDE2A，PDGFRB，PDLIM3，PECAM1，PLEKHO1，PLPP4，PODN，PRELP，PTGIS，PTPRM，PXDN，RAB31，RAB34，RFTN1，RHOJ，RTL8B，S1PR1，SERPINF1，SERPING1，SFRP2，SFRP4，SHISAL1，SLC22A17，SLC24A3，SLIT3，SPARC，SPARCL1，SPOCK1，SSC5D，SULF1，SYDE1，SYNC，SYNDIG1，SYT11，TAFA5，TAGLN，TEK，TGFB3，THBS2，THY1，TIE1，TIMP2，TMEM119，TMEM200B，TNS1，TSHZ3，TSPYL5，TUBB6，TWIST2，VASH1，VCAN，VEGFC，VIM，VSTM4，ZEB2，ZNF469
